# The complete mitochondrial genome of the pear pyralid moth, *Euzophera pyriella* Yang

**DOI:** 10.1080/23802359.2017.1325338

**Published:** 2017-05-12

**Authors:** Minglu Yang, Shiqian Feng, Yu Cao, Xu Han, Renci Xiong, Zhihong Li

**Affiliations:** a College of Plant Science, Tarim University, Alar, China;; b Department of Entomology, College of Plant Protection, China Agricultural University, Beijing, China

**Keywords:** Mitochondrial genome, *Euzophera*, Pyralidae, *Euzophera pyriella* Yang

## Abstract

The pear pyralid moth, *Euzophera pyriella* Yang, is an important fruit pest in Xinjiang, China. Here, we report the complete mitochondrial genome of *E. pyriella* which was 15,184 bp and composed of 13 protein-coding genes, 22 tRNA genes, 2 rRNA genes, and a control region. 21 genes were in majority strand and 16 genes were in minority strand. ATG, ATT, CGA, GAT were initiation codons and TAA, TA, T were termination codons. The tRNA genes ranged in length from 63 to 72 bp. The length of 12S and 16S rRNA genes were 773 and 1330 bp, respectively. The species belong to the family Pyralidae and have a closer relationship according to the phylogenetic analyses.

The pear pyralid moth, *Euzophera pyriella* Yang, was firstly reported in China in 1994 (Yang 1994). This is a major pest in Xinjiang pear (*Pyrus sinkiangensis*), which is of important economic value and mainly distributed in Xinjiang Uygur Autonomous Region in the northwest of China (Hou et al. 2011). The animal mitochondrial (mt) genomes are typically a single circular chromosome, ∼16 kb in size, and contain 37 genes: 13 protein-coding genes, 2 ribosomal RNA (rRNA) genes, and 22 transfer RNA (tRNA) genes (Boore 1999; Lavrov 2007).

The samples of *Euzophera pyriella* Yang were collected in a pear garden (81.30E, 40.54N) in Aral, Xinjiang Uygur Autonomous Region, China, and stored in the insect specimen room of Tarim University with an accession number XLYBM01. Total DNA was extracted from one male/female *E. pyriella* using Ezup Column Animal Genomic DNA Purification Kit (Shanghai, China). The partial CO1 sequence was used as the base to get the complete mt genome sequence through primer walking. All PCR primers were synthetized and all PCR products were sequenced by Sangon Biotech (Shanghai, China).

The complete mitochondrial genome sequence of *E. pyriella* (15,184 bp) was composed of 37.8% A, 42% T, 12.5% C, 7.6% G, with an extremely lower GC content (20.2%). The mt genome consists of 13 protein-coding genes, 2 rRNA genes, and 22 tRNA genes (Figure 1). 21 genes were in majority strand while 16 genes were in minority strand. ATG, ATT, CGA, GAT were initiation codons, TAA, TA, T termination codons. The tRNA genes ranged in size from 63 to (tRNA Arginine) 72 bp (tRNA Serine). The length of 12S and 16S rRNA genes were 773 and 1330 bp, respectively, which were located between the tRNA Serine gene and D-loop and separated by tRNA Valine. Analysing the phylogenetic relationships, we found that all species belong to Pyralidae forming a monophyly that have a closer relationship with each other.

**Figure 1.**
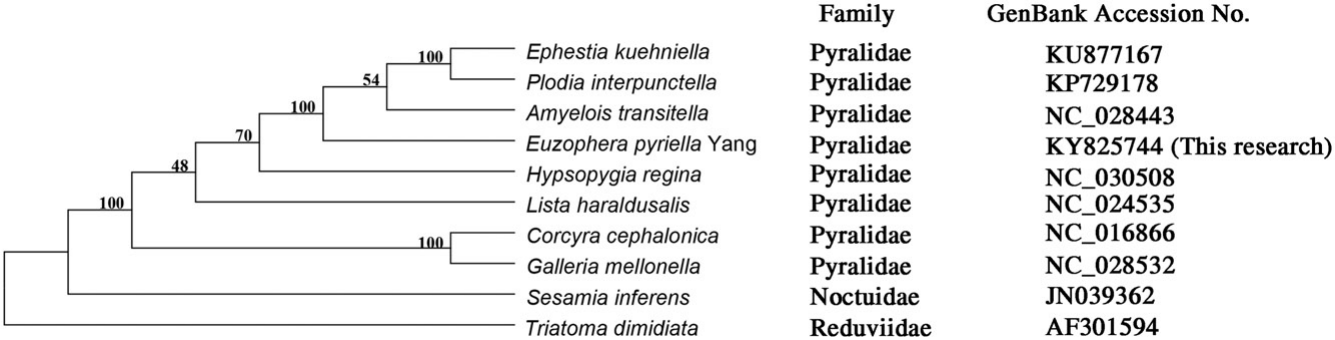
A phylogenetic tree based on whole mitochondrial genome sequences of 10 species. 8 species belong to Pyralidae; 1 species belongs to Noctuidae. *Triatoma dimidiate* (Hemiptera: Reduviidae) was used as an outgroup.
